# Compartmentalized cAMP signalling and control of cardiac rhythm

**DOI:** 10.1098/rstb.2022.0172

**Published:** 2023-06-19

**Authors:** Jakub Tomek, Manuela Zaccolo

**Affiliations:** ^1^ Department of Physiology, Anatomy and Genetics, University of Oxford, Parks Road, Oxford OX1 3PT, UK; ^2^ Oxford NIHR Biomedical Research Centre, University of Oxford, Parks Road, Oxford OX1 3PT, UK

**Keywords:** cAMP, PKA, pacemaker, heart rhythm, compartmentalized signalling

## Abstract

In the last 30 years, the field of cyclic adenosine 3′,5′-monophosphate (cAMP) signalling has witnessed a transformative development with the realization that cAMP is compartmentalized and that spatial regulation of cAMP is critical for faithful signal propagation and hormonal specificity. This recognition has changed our understanding of cAMP signalling from the canonical model, where a linear pathway connects a plasma membrane receptor to intracellular effectors and their targets, to a model where signal transduction occurs within a complex network of alternative branches and where an individual receptor leads to activation of a limited fraction of the network, enabled by local regulation of independent signalling units, resulting in a specific functional outcome. The cardiac myocyte has served as the cell model for many of the original findings leading to this paradigm. In this review, we cover some of the evidence supporting this new perspective and discuss how this model is providing novel mechanistic insight into cardiac myocyte physiology. With a focus on the regulation of cardiac rhythm, we consider how this model can provide an original framework for the identification of disease mechanisms.

This article is part of the theme issue ‘The heartbeat: its molecular basis and physiological mechanisms’.

## 1. Introduction

Activation of the cardiac sympathetic nervous system underlies the ‘fight-or-flight’ response to stress or exercise, increasing heart rate, contractility and the rate of cardiac relaxation [1]. The effect is to enhance cardiac output and to enable the heart to meet the body's increased demand for the delivery of oxygenated blood. The signal to the heart is conveyed by catecholamines that act on β-adrenergic receptors (ARs) in cardiomyocytes: norepinephrine (released mainly by the sympathetic nerves in the heart) and epinephrine (released by the adrenal medulla and delivered via the bloodstream). β-ARs are Gs protein coupled receptors (GPCRs) that, when activated, trigger an intricate and finely tuned network of intracellular signalling proteins and molecules that result in the required cardiac myocyte response.

Cyclic adenosine 3′,5′-monophosphate (cAMP) is the second messenger that transduces the extracellular sympathetic signal to intracellular protein effectors. Recent studies have clearly demonstrated that signalling mediated by cAMP does not involve a global, homogeneous increase in the intracellular concentration of the second messenger. Instead, cAMP operates through locally regulated nanodomains located at multiple subcellular sites, each uniquely associated with a range of distinct effectors, regulators and downstream targets [2]. The targets activated by cAMP include membrane channels and receptors, proteins involved in calcium cycling or contractility, but also proteins involved in cell survival, regulation of metabolism, control of gene expression and many others [3]. The multiplicity of targets that can be activated by cAMP raises the question of how a specific stimulus triggers only the required response. cAMP signalling compartmentalization provides an effective solution as, through the organization of multiprotein complexes (or signalosomes) where the molecular elements involved in signal transduction congregate at defined subcellular locations, the disparate intracellular targets of cAMP can be controlled with a large degree of independence [4]. A recent study reported that activation of different GPCRs, including β-ARs, by low doses of agonist can generate a highly compartmentalized cAMP signal in the immediate vicinity of the receptor. These localized pools of cAMP, which the authors name receptor-associated independent cAMP nanodomains (RAINs), are not affected by cAMP originating from a different receptor or by cytosolic cAMP and extend by only a few tens of nanometres away from the receptor that triggers them, providing evidence that the cell can operate simultaneously multiple signals generated by different stimuli while maintaining signal specificity [5].

Here, we provide an overview of how distinct elements of the cAMP signalling cascade are distinctively organized in local signalling units that alone, or in coordination with other units, orchestrate the physiological activity of the cardiomyocyte. Most of the experimental work leading to the notion of cAMP signalling compartmentalization has been conducted in ventricular myocytes obtained from animal models; however, data are emerging that support a similar organization in atrial and conduction system myocytes and in human cardiac myocytes. In this review, we focus on the regulation of cardiac rhythm, and we discuss how unravelling the details of this organization is necessary to better understand the molecular basis of cardiac rhythm disturbances.

## 2. An overview of cAMP signalling in the cardiac myocyte

**Figure 1.**
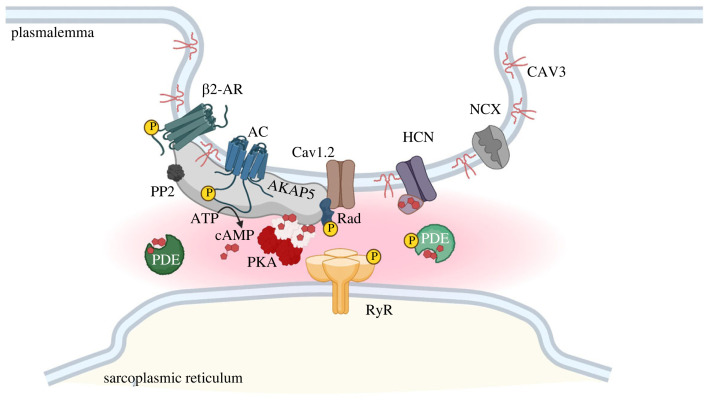
Illustration of a cAMP nanodomain localized at the plasmalemma. *β*2-AR, beta2 adrenergic receptor; AC, adenylyl cyclase; Cav1.2, voltage-dependent: L type calcium channel, alpha 1C subunit; HCN, hyperpolarization, cyclic nucleotide gated ion channel; NCX, sodium/calcium exchanger; AKAP5, A kinase anchoring protein 5; Rad, small Ras-like G protein, inhibitor of calcium channels; PP2, protein phosphatase type 2; PDE, phosphodiesterase; PKA, protein kinase A; RyR, ryanodine receptor. CAV3, caveolin-3. Yellow symbols circling a P represent phosphorylation by PKA. Shaded area between caveolar membrane and sarcoplasmic reticulum membrane indicates confined increase in cAMP. (Online version in red colour.)

The general sequence of events in the intracellular response to β-adrenergic stimulation is as follows: a β-AR in the membrane signals to a functionally coupled protein, termed adenylyl cyclase (AC), that produces cAMP. The cAMP molecules then reach and activate one of the effector proteins, such as protein kinase A (PKA), which modulate a range of components of intracellular machinery via phosphorylation. The cAMP molecules produced in the cell are degraded by proteins termed phosphodiesterases (PDEs), which help maintaining different cAMP concentrations in different parts of the cells and target phosphorylation is reversed by the activity of phosphatases. The proteins involved in this chain of events are not dispersed freely at the plasma membrane or in the cytosol but are anchored to scaffolding proteins that organize, via protein–protein interactions, local signalosomes (figure 1). The scaffolding proteins often belong to the family of A kinase anchoring proteins (AKAPs), so defined on the basis of their ability to interact, through an amphipathic helix, with the PKA regulatory subunit dimerization and docking domain [6]. Apart from the PKA anchoring site, AKAPs show no sequence similarity and different members of this family localize to distinct subcellular locations, where they tether PKA in proximity to one or more of its phosphorylation targets. In addition to PKA, AKAPs may bind ACs, PDEs, that can locally degrade cAMP, and phosphatases, that can dephosphorylate the signal delivered by PKA. Thus AKAPs provide a platform for tight spatio-temporal regulation of the cAMP signalling cascade, where signal generation, signal delivery to the appropriate target and signal extinction can occur locally [2]. Below, we discuss in greater detail the role that the different components of the cAMP pathway play in cardiac myocytes.

## 3. Beta-adrenergic receptors

The two main subtypes of the β-AR expressed in the heart are *β*1 (approx. 80%) and *β*2 (approx. 20%) [7], although *β*3 was also described [8]. *β*1-AR stimulation leads to the highest increase in contractility and heart rate, but chronic activation is deleterious to the heart, promoting cardiac remodelling and transition to heart failure with reduced ejection fraction [9,10]. Conversely, the activation of the *β*2-AR appears to be cardioprotective, while also partly increasing cardiac contractility [11,12].

Experiments in mice and rats indicate that the two main subtypes are differently localized within a cardiac myocyte: while *β*1 was found to be present through the membrane in t-tubules and the membrane crest, *β*2 was found to be mainly in the t-tubules in the healthy heart [13–15]. Another difference between the receptor subtypes is that *β*2-induced cAMP signals appear to be relatively local and constrained near the cellular membrane in healthy cells, whereas *β*1-stimulation leads to generation of cAMP signals that is far-reaching [16,17].

Compartmentalization of β-ARs has been reported also to occur in sinoatrial node (SAN) cells, the pacemaker site for the heart, where *β*2 rather than *β*1 receptors concentrate predominantly within caveolar structures where they associate with pacemaker channels and preferentially regulate their activity [18,19].

## 4. Adenylyl cyclases

ACs are the enzymes that synthesize cAMP from ATP and most of the cardiac ACs are bound to the membrane, where they can form complexes with β-ARs. There are nine membrane-bound isoforms (AC1-9) and one so-called soluble isoform (AC10), which does not respond to extracellular β-adrenergic stimulation but is activated by bicarbonate and calcium. AC1–9 isoforms are activated by G*α*s and inhibited by G*α*i and are differentially regulated by a number of mechanisms, including interaction with calcium-calmodulin, interaction with G*βγ*, phosphorylation by PKA, protein kinase C (PKC) or calcium calmodulin-activated kinase II (CaMKII) [20]. The diversity of the regulatory modalities these enzymes display suggests that different AC isoforms may serve different functions. In support of this notion, the distribution of AC isoforms differs between SAN cells and contractile myocytes. The two main types of ACs expressed in working myocytes are AC5 and AC6, which are Ca^2+^ inhibited [13,21]. AC5 is present predominantly in the t-tubules, where it can associate with both *β*1 and *β*2-ARs [22]. Conversely, AC6 is present mainly in the crest of the myocyte plasmalemma (outside t-tubules), where it may associate with *β*1-ARs [22].

By contrast, the Ca^2+^-activated AC1 is the prevalent isoforms expressed in SAN cell [23], where it plays an important role in the sympathetic control of heart rate, as discussed below. SAN cells also express AC8, another Ca^2+^ activated enzyme [24]. The observation that cardiac-specific overexpression of AC8 results in increased heart rate and attenuates heart rate variability in the absence of adrenergic stimulation, has led to the suggestion that this isoform may be critical for sympathetic-independent regulation of SAN automaticity [24].

AC9, a calcium-calmodulin inhibited isoform, represents only a relatively minor fraction of overall AC activity in the heart but it was found to be crucial for sympathetic regulation of the slow delayed potassium rectifier current I_Ks_ [25,26], being part of a plasmalemma complex that includes the KCNQ1 component of I_ks_ channels and the AKAP Yotiao [26]. AC9, therefore, appears to be an important source of repolarization reserve, and thus cardioprotection, in mammals such as humans, dogs or rabbits, where I_Ks_ is a key repolarization current during β-adrenergic stimulation. AC9 has also been reported to regulate cardiac contraction rate in mice [27] through its action at an apparently distinct signalling domain that involves the scaffolding protein, Popeye domain containing protein 1 (POPDC1) and the two-pore-domain potassium channel TREK-1 [28].

The signal transduction between β-ARs and ACs involves the activation of G*α*_s_-proteins, which stimulate the production of cAMP, and G*α*_i_, which inhibit it. All β-ARs were observed to be coupled to G*α*_s_, but *β*_2_ was also found to be concurrently coupled to G*α*_i_. To an extent, the coupling between a receptor and these G-proteins is dynamic and may change depending on the specific conditions the cell is in [29].

## 5. cAMP effectors

The cAMP molecules produced by ACs activate various effector proteins. Some effectors can be local to the AC producing cAMP, both being bound to the same AKAP, while some are more distal, relying on cAMP diffusion from the ACs. The extent to which cAMP can diffuse away from the site of synthesis and activate distal effectors is likely to depend on the specific GPCR, on the concentration and/or duration of the stimulus and on the PDE hydrolytic activity associated with that specific receptor. For example, subnanomolar concentrations of the hormone GLP-1 were shown to generate a pool of cAMP radiating only about 30 nm away from the receptor at the plasma membrane, and this restricted diffusion was abolished when the PDEs were inhibited [5].

The known cAMP effector proteins are PKA [30], exchange protein directly activated by cAMP (EPAC) [31], POPDCs [32] and cyclic hyperpolarization-activated, nucleotide-gated (HCN) channels [33].

In the cardiac myocyte, PKA phosphorylates a wide range of targets, see [30] for an overview. Key proteins in myocyte calcium handling are phosphorylated by PKA, adapting the heart to the need for rapid and vigorous cardiac contraction and relaxation during stress responses. These include the L-type calcium channels, phospholamban (PLB, disinhibiting SERCA pumps and increasing calcium uptake into the sarcoplasmic reticulum, SR), and myofilament proteins involved in contractility, such as troponin I and myosin binding protein C [34]. Several of the proteins phosphorylated by PKA are also heavily involved in the generation of heart rhythm as discussed in more detail below.

Beyond the control of excitation-contraction coupling and heart rate, PKA signalling in the nucleus can promote cell hypertrophy via processes involving CREB-mediated transcriptional changes; however, cytoplasmic PKA activity appears anti-hypertrophic [30]. PKA signalling also contributes to myocardial damage during reperfusion that follows ischaemia [30]. One aspect of PKA function that is worth keeping in mind is that it can be also activated by other means than by cAMP binding. For example, in the case of ischaemia-induced activation, PKA is at least in part activated by reduced expression of the RIα inhibitory subunit of PKA, a consequence of increased reactive oxygen species generated during ischaemia [35].

Finally, an important feature of PKA activity is that it signals back to the machinery involved in the production of cAMP, by phosphorylating the β-ARs and AC5/6 [36,37], thus contributing to an autoinhibitory feedback loop during sustained stimulation. The *β*2-AR may undergo a switch from coupling with G*α*_s_ to coupling with G*α*_i_ [38,39], inhibiting cAMP production. The *β*1-AR may become uncoupled from the G*α*_s_ protein by PKA activity [13,40], but the possibility of G*α*_s_ to G*α*_i_ switch was also suggested [41]. PKA can also phosphorylate and activate PDE3 [42], PDE4 [43] and PDE8 [44] isoforms, increasing their ability to hydrolyse cAMP, as well as phosphatase regulatory elements, leading to either activation or inhibition of phosphatase activity [45], again triggering complex feed-back loops. Together, these properties of PKA give an indication of the complexity and locality-dependence of the cAMP signalling cascade.

EPAC is another prominent cAMP effector that has been implicated in multiple areas of cardiomyocyte regulation, such as modulation of ionic currents, promotion of myocardial hypertrophy and fibrosis, gene transcription, pro-apoptotic signalling [46], as well as calcium handling and signalling [47].

The POPDC proteins are more recently described effectors of cAMP. These transmembrane proteins are expressed as three isoforms (POPDC1-3) and POPDC1 and POPDC2 and are highly expressed in the heart, including in cells of the conduction system. Loss-of-function mutations in animal models results in defective adaptation of heart rate to stress and patients carrying missense mutations in POPDC genes present with arrhythmias, heavily implicating POPDC proteins in the control of pacemaking activity [32].

HCN channels include the channels carrying the pacemaking ‘funny’ current I_f_ which contributes to the spontaneous diastolic depolarization of SAN cells. Direct binding of cAMP to HCN channels increases their open probability [48]. HCN4 is the predominant isoform expressed in rodent SAN cells, whereas HCN1, HCN2 and HCN4 are all highly expressed in human SAN [49].

## 6. Phosphodiesterases

**Figure 2.**
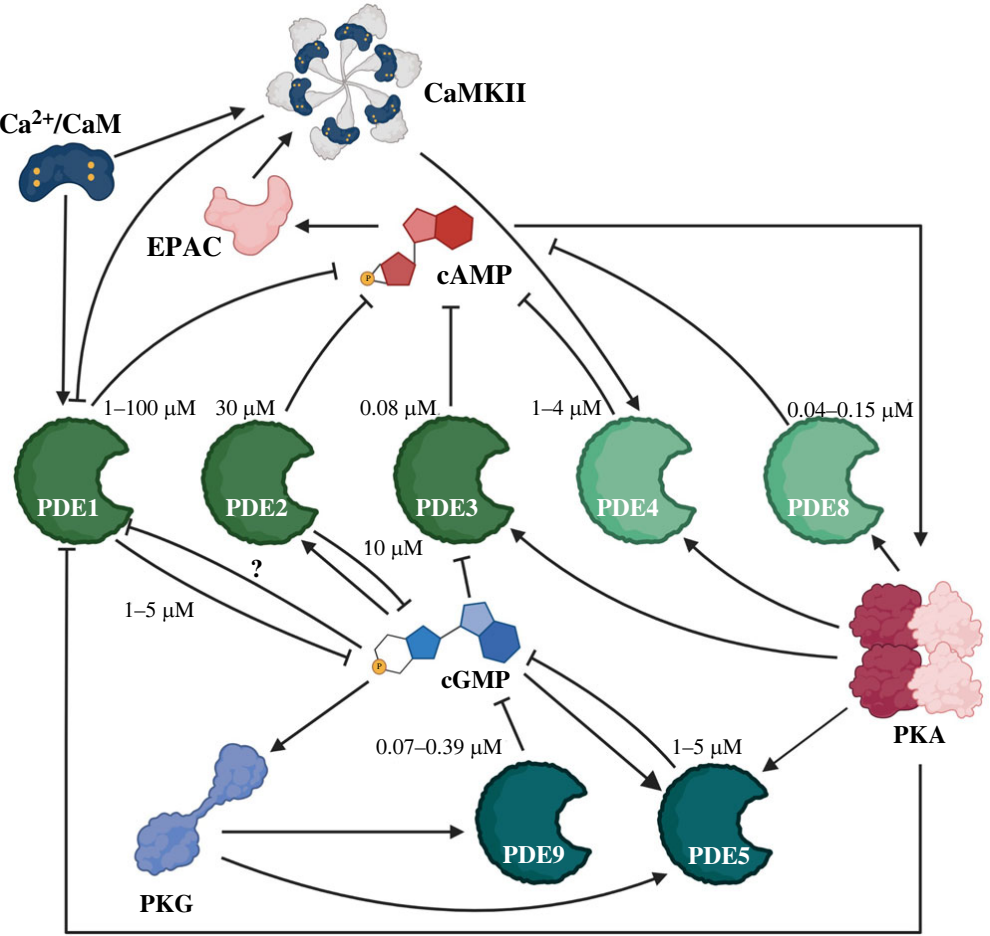
Diagram illustrating the complex regulatory network involving phosphodiesterases, cAMP, cGMP and calcium. Ca2+/CaM, calcium/calmodulin; CamKII, calcium/calmodulin activated kinase II; EPAC, exchange factor activated by PKA; PKG, protein kinase G; PKA, protein kinase A; PDE, phosphodiesterase. Dark green indicates cGMP-selective PDEs. Lighter green indicates dual-specificity PDEs. Very light green indicates cAMP-selective PDEs. The affinity of binding of the PDE with the cyclic nucleotide is indicated. (Online version in colour.)

The cyclic nucleotide PDEs are enzymes that hydrolyse cAMP and cyclic guanosine monophosphate (cGMP) and play a key role in shaping the intracellular concentration gradients of these second messengers. By being localized to specific subcellular site, the PDEs determine the local concentration of cyclic nucleotide at individual signalosomes, effectively determining which targets are phosphorylated. PDEs can selectively hydrolyse cAMP or cGMP, while some isoforms can hydrolyse both second messengers. cGMP is produced by guanyl cyclases (in response to nitric oxide and natriuretic peptides) and often has opposing effects to those of cAMP [50]. The most prominent PDE families expressed in the heart are PDE1–5, 8 and 9. These families differ in their selectivity for the cyclic nucleotides and in how their enzymatic activity is modulated, resulting in a complex network of cross-talk regulation (a visual summary is provided in figure 2). Each PDE family may include several variants that differ in affinity for the cyclic nucleotides and in their subcellular localization. When considering the role of individual PDEs, affinity for substrate, level of expression and localization should be considered in conjunction. PDE isoform with low affinity for cAMP or cGMP, or isoforms that are expressed at low levels, may still play a prominent role in the response to a certain stimulus if the enzyme is localized in a compartment where the concentration of the second messenger reaches a sufficiently elevated concentration, despite a minimal, or even undetectable, global concentration change. Below we briefly introduce the main cardiac PDE families, with more comprehensive overview available in [51–53].

The PDE1 family includes the PDE1A1, PDE1A2 and PDE1B1 isoforms [54] and hydrolyses both cAMP and cGMP, with most variants having a greater affinity for cGMP [55]. PDE1 isoforms are stimulated by calcium and calmodulin, while being inhibited by PKA and CamKII phosphorylation [50,56,57]. PDE1 activity was linked to pro-hypertrophic signalling in rats [58], with recent studies in humans and dogs demonstrating that PDE1 blockade increases contractile performance [59,60]. A possible explanation for the observed increased contractility was reported by Muller *et al*. [61], who demonstrated that the L-type calcium current, the trigger to calcium-sensitive calcium release, is potentiated by PDE1 inhibition. Interestingly, the study found only relatively small changes at PKA activity sites distal to L-type channels, such as PLB or the contractile machinery, indicating the importance of PDE1 in local control of cAMP.

PDE2A also hydrolyses both cAMP and cGMP, with a slightly higher affinity for cGMP, and is expressed in cardiac myocytes as PDE2A1 and PDE2A2 isoforms, whereas expression of the PDE2A3 isoform appears to be limited to the brain, at least in the mouse [62]. Notably, PDE2A is potently stimulated by cGMP, which leads to an increase in the PDE's affinity for cAMP [50]. This makes PDE2A an important element in the cGMP-cAMP crosstalk, where a cGMP increase may attenuate cAMP signalling at specific locations.

PDE3, the predominant cAMP-hydrolysing family in the human heart [63] is expressed in cardiac myocytes as PDE3A1, PDE3A2 and PDE3B isoforms that are inhibited by cGMP, presenting another important element of the cAMP-cGMP crosstalk [64]. PDE3A is also phosphorylated and activated by PKA, further contributing to feedback loop regulation [65]. PDE3A isoforms are major regulators of cardiac contractility under β-adrenergic stimulation, as they contribute to the control of cAMP levels near the SERCA pumps in the mouse and humans [66,67] via interaction with AKAP18 [67,68]. In addition, multiple studies indicate that PDE3A regulates cAMP levels in the vicinity of channels mediating the L-type calcium current, but the results are highly species- and study-dependent. For example, PDE3 inhibition was observed to potently increase L-type calcium current in mouse SAN cells, but not right atrial myocytes [69]. At the same time, PDE3 inhibition was reported to lead to a substantial increase in L-type calcium current in human atrial cells [70]. In the ventricles of guinea pigs, PDE3A was shown to regulate the cAMP pool associated with the regulation of L-type calcium current [61,71], and this interaction was also suggested in one study using mouse cells [72], although this finding was not confirmed in another study [67]. The other type of PDE3, PDE3B, is localized preferentially in the t-tubules, close to dyads and mitochondria, and is thought to partly mediate the myocardial damage during ischaemia-reperfusion and probably contributes to the regulation of cellular metabolism [73].

PDE4 is a complex family including more than 20 variants that hydrolyse exclusively cAMP [74]. Although PDE4 activity is more prominent in rodent than in human hearts, isoforms of the PDE4A, PDE4 and PDE4D families are also expressed in human cardiac tissue [75]. Like PDE3, some PDE4 isoforms are activated by PKA and act as important modulators of myocyte electrophysiology and calcium handling and are thus of interest in the context of cardiac rhythm control. Different PDE4 isoforms play distinct roles in the cell and are localized differently [76]. For example, PDE4B has been implicated, together with PDE4D, in the regulation of cAMP that influences the function of L-type calcium channels [77,78]. PDE4D3 regulates the slowly activating potassium channel I_Ks_ and PDE4D co-regulates PLB, and thus SERCA function, together with PDE3A [76]. PDE4D3 was suggested to regulate the phosphorylation of ryanodine receptors by PKA [76]; however, the nature and impact of such phosphorylation is controversial [79]. In addition, PDE4 is involved in the regulation of β-receptor desensitization [80]. Finally, in neonatal rat myocytes, PDE4D was shown to be a central factor determining the ability of *β*1- and *β*2-AR signalling to generate a compartmentalized cAMP signal with a distinct range of action. As mentioned above, compared to *β*1-AR, the activation of *β*2-AR results in a much more restricted signal that does not regulate distal sites such as phospholamban or contractile apparatus, but this restriction was lost in the presence of PDE4 inhibitors, highlighting the role of PDE4 in shaping the localized nature of cAMP signalling within the cell [81].

A synergistic action of multiple PDEs has been described at certain subcellular locations. For example, concomitant inhibition of PDE3 and PDE4 results in more than additive effect on the phosphorylation of L-type calcium channels or PLB, which are targets under the regulation of both enzymes [82–84]. It is possible that the higher-cAMP-affinity PDE3 is typically the main cAMP-degrading PDE in these nanodomains, but its blockade leads to a local increase in cAMP, which then activates (via PKA) the lower-affinity PDE4 to increase its cAMP-degrading performance, maintaining the cAMP concentration at a relatively controlled level [85].

An important feature of the cAMP-hydrolysing PDE1–4 is that there are pronounced species differences in the extent of their contribution to cAMP degradation. In mice and rats, PDE4 is the dominant PDE family, whereas it is less important in larger mammals [51], and is comparably marginal in humans, where it contributes less than 10% of total cardiac PDE activity [75]. PDE4 isoforms have been reported to contribute approximately 15% of total PDE activity in human atrial myocytes with PDE4D playing a critical role in the regulation of L-type calcium channels in these cells [78]. Interestingly, a study directly comparing PDE activity between humans, rats and mice reported that the relative decrease in PDE4 prominence is not owing to lower PDE4 activity (which is in fact similar between the species), but results from a much higher activity of PDE1–3 in humans versus rats [75]. One functional implication of this difference is that PDE4 inhibition in small rodents leads to global changes in cAMP signalling, given that other PDEs have a limited impact on dissipating cAMP gradients, and results in the activation of targets distal to the site of cAMP production. Conversely, PDE4 inhibition in humans leads only to local cAMP changes under direct control of PDE4, as the global cAMP signalling is prevented by the action of strong PDE1/PDE3 activity. Such species differences are important, e.g. when interpreting studies on how promising blockers of distinct PDEs are, as blockers of PDE4 are likely to have a stronger and more generalized effect in mice and rats than they might in humans. Furthermore, a very recent study reported marked sex-differences in regional cAMP degradation dynamics in murine hearts, and these could be traced to differential activity of PDEs and PDE4D expression [86].

PDE8 is another cAMP-specific PDE expressed in the heart as PDE8A and PDE8B isoforms. Studies on this family are limited but its role appears to be linked to the regulation of excitation contraction coupling and ryanodine receptor activity, although PDE8A-/- mice show no cardiac phenotype. Deletion of the PDE8A gene also does not result in overall increased PKA activity in cardiac myocytes, suggesting a role confined in a specific compartment [87]. In a recent study conducted in human atrial myocytes, PDE8B was found to localize at the plasmalemma in close proximity to the L-type calcium channel where it regulates PKA-dependent phosphorylation of the channel. The study also found upregulation of PDE8B in persistent atrial fibrillation, thus providing a potential mechanism for the proarrhythmic reduction in I_Ca,L_ observed in this condition [88].

PDE5 and PDE9 are cardiac PDEs that, despite being cGMP-selective, are relevant to cAMP signalling in that they can contribute to the cAMP/cGMP cross-talk described above. PDE5 inhibition has been shown to protect the heart from ischemic stress and attenuate hypertrophy and heart failure remodelling in animal models [53]. PDE5 also contributes to the regulation of calcium handling by interacting with other PDEs and may limit the response of myocytes to β-AR stimulation. Specifically, it has been observed in murine sinoatrial cells that PDE5 inhibition attenuates the beating rate increase following sympathetic stimulation, but only if PDE2 function is intact [89]. This can be explained by the fact that PDE5 inhibition increases cGMP concentration in the same compartment where the cGMP-activated PDE2 is present. cGMP subsequently activates PDE2, limiting cAMP concentrations in its vicinity, thus counteracting the effect of β-AR activation.

PDE9 is expressed, although at very low level, and is functional in the heart in several species, including humans and it appears to selectively degrade cGMP generated on activation of natriuretic peptide receptors. Its expression has been reported to be upregulated in heart failure and its inhibition has been suggested as a potential treatment for this condition [90].

## 7. Phosphatases

Serine/threonine phosphatases, which dephosphorylate PKA targets, are also often present in cAMP signalosomes and, together with PDEs, provide a key mechanism to regulate the cAMP signal locally. The fact that different phosphatases associate with individual signalosomes provides further complexity to the regulation of cAMP signals by enabling additional local variation in the signal dynamics. In the heart, protein phosphatase 1 (PP1) and protein phosphatase 2A (PP2A) catalyse between 70% and 90% of all dephosphorylation events [91], although protein phosphatase 2B (PP2B) (calcineurin, CaN) also contributes [92]. The number of phosphatases is significantly smaller than the number of targets they dephosphorylate and substrate specificity primarily relies on the role of regulatory subunit, which also dictate their subcellular localization [93].

In cardiac myocytes, PP1 and PP2A dephosphorylate several PKA targets, including the Cav1.2 subunit of the L-type calcium channel, PLB, phospolemman (which regulates the activity of the Na^+^/K^+^ ATPase) and the KCNQ1 channel. The latter forms a complex with PKA, PP1 and the AKAP Yotiao, which regulates the channel phosphorylation on β-AR stimulation [94]. Both PP1 and PP2A have also been found to co-immunoprecipitate with RyR2 and mAKAP and have been shown to dephosphorylate PKA targets at the thin filament (troponin I and myosin binding protein C). CaN also has been shown to interact with multiple AKAPs in the cardiac myocyte, including the plasmalemma anchored AKAP5 [95], the Z-line localized AKAP Cypher/Zasp [96], the mitochondrial AKAP1 [97] and mAKAP at the nuclear envelope [98]. Currently, limited information is available on the specific role of phosphatases in the regulation of β-AR signalling in SAN cells, although both PP1 [99] and PP2A [100] have been implicated. A significant basal phosphatase activity has been proposed to act in concert with PDEs activity to maintain a low phosphorylation level of clock molecules (see below) in the resting state, thus enabling a rapid, robust response when the β-ARs are activated.

## 8. Scaffolding proteins

Scaffolding proteins play an important part in the compartmentalization of cAMP signalling as they organize the multiprotein complexes that operate as distinct signalosomes. The AKAPs facilitate control by bringing together, at defined locations, regulators of the cAMP pathway, including GPCR, ACs, downstream effectors of cAMP and their targets, as well as PDEs and phosphatases, in different assortment depending on the specific signalosome. More than 14 AKAPs have been described in the heart, including AKAP5 (also termed AKAP150/79) which regulates L-type channels [101] AKAP7 (also termed AKAP18) that regulates PLB and thus SERCA pumps and also interacts with CamKII [67,68], AKAP9 (also called Yotiao), involved in the regulation of the slow repolarization current I_Ks_ [26,94], AKAP12 (or gravin), AKAP-Lbc and mAKAP [102]. Although specific information on AKAPs expression in SAN cells is scarce, these proteins are likely to play an important role in coordinating local control of cAMP signalling, as observed in working myocytes. Interestingly, genetic ablation of AKAP5 in mice with long QT syndrome 8, a condition characterized by sinus bradycardia, prolonged QT interval and lethal arrhythmias, was found to restore normal gating of Cav1.2 channels and to protect from arrhythmia [103], and AKAP10 (D-AKAP2) has been implicated in the control of cardiac rhythm both in mice and in humans [104].

Other scaffolding proteins are relevant for the coordination of local cAMP signalling with the machinery involved in pacemaking. A prominent example is caveolin-3 (CAV3), an integral membrane protein that is essential in the formation of caveolae (figure 1). These are invaginations of the SAN cell plasmalemma where multiple elements of the cAMP signalling pathway congregate together with multiple elements that are essential for the functional integrity of the pacemaker activity [105]. Several studies show interaction of CAV3 with HCN4 and disruption of caveolae with methyl-β-cyclodestrin ablates the ability of *β*2-ARs to modulate HCN4 activity, supporting their co-localization at the membrane [106]. Proximity of CAV3 with L-type calcium channels and ryanodine receptors has also been reported [107] as well as interaction of CAV3 with the sodium-calcium exchanger (NCX) [108]. Finally, AKAP5, which forms a complex with AC5/6, PKA, PP2 and Cav1.2, also interact with CAV3, although this complex has been demonstrated only in ventricular myocytes so far [109].

## 9. cAMP and the control of heart rhythm

Normal cardiac rhythm is generated in the SAN by periodic spontaneous depolarization of SAN cells. The activation signal then travels to the atria and the atrioventricular node, where it is delayed, providing time for the ventricles to fill with blood, and is then transmitted to the ventricles via the bundle of His and Purkinje fibres.^1^ The SAN is extensively innervated by both sympathetic and parasympathetic nerves, which increase and reduce, respectively, the heart rate to match the physiological needs of the body. The sympathetic control of heart rate mediated by cAMP signalling is relatively complex and there are important differences with cAMP signalling in ventricular myocytes. The primary physiological parameter modulated by cAMP signalling in the SAN cells is the period of spontaneous activation, as opposed to contractility and conduction properties, as is the case for working cardiac myocytes.

The overall cAMP levels appear to be three times higher in SAN than in ventricles, despite the concurrent higher activity of PDEs, and this is a result of constitutive AC activity, rather than β-AR hyperactivation [85]. The calcium-activated AC1 is a crucial source of cAMP in SAN cells, whereas it contributes only relatively marginally to cAMP production in the ventricles [23,111], which can explain the elevated cAMP levels. AC1 is co-localized within caveolae with proteins involved in the generation of cardiac rhythm, including HCN channels underlying the funny current, ryanodine receptors, and CaV channels underlying the L-type calcium current [111]. The calcium-activated nature of AC1 leads to a self-sustaining feedforward loop, where high cAMP activity activates and accelerates cellular calcium handling, which in turn stimulates cAMP formation by the AC1. This process can explain the sustained automaticity of the SAN cells in response to β-adrenergic stimulation.

**Figure 3.**
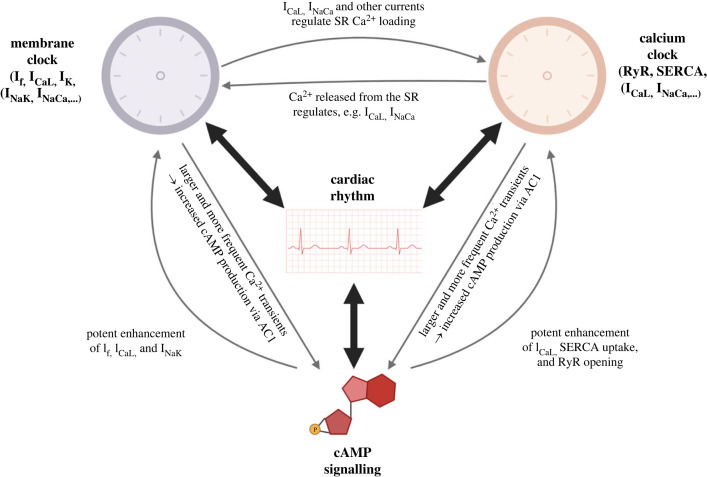
A graphical overview of how the two rhythm-generating clocks interact with cAMP signalling to generate cardiac rhythm. I_f_, funny current generated by HCN channels; I_CaL_, L-type calcium current; I_K_, repolarizing potassium currents; I_NaK_, current generated by the sodium-potassium pump; I_NaCa_, current generated by the sodium-calcium exchanger; SR, sarcoplasmic reticulum; RyR, ryanodine receptors; SERCA, SERCA pumps in the sarcoplasmic reticulum; AC1, adenylyl cyclase 1. Any resemblance of the diagram to a flux capacitor is purely coincidental. (Online version in colour.)

The rhythm generation in SAN cells is relatively complex even without considering cAMP signalling. The contributors to the periodic depolarization are typically described as members of two ‘clocks’ [85,112] and their interaction with cAMP signalling is summarized in figure 3. The ‘membrane clock’ consists of a periodic sequence of activation of various ionic currents, such as the funny current I_f_, L-type calcium current, the current mediated by the NCX, and repolarizing currents, such as potassium currents and the current mediated by the sodium-potassium pump. In essence, a prototypical cycle of the membrane clock is as follows: I_f_ gradually depolarizes the cell, eventually activating the L-type channels and, with the contribution of NCX, an action potential is generated. The repolarizing currents then bring the membrane potential down, preparing the membrane clock for another cycle. The second clock is termed the ‘calcium clock’, where calcium may be released spontaneously from the SR via ryanodine receptors; this is promoted both by elevated calcium levels in the cytosol and within the SR. A typical cycle of the calcium clock is as follows: during quiescence, calcium is reuptaken by SERCA pumps. When a high-enough concentration of calcium in the SR is achieved, the leak of calcium ions through ryanodine receptors increases, eventually leading to a situation where sufficient calcium is present outside the SR, as well as inside, causing a spontaneous calcium release from the SR. Part of the calcium released is reuptaken in the SR via SERCA pumps, but a part is exported from the cell by NCX, which in the process depolarizes the cell, given the electrogenic nature of NCX (exchanging 1 calcium for 3 sodium ions), providing a second source of periodic depolarization.

The two clocks are tightly coupled. L-type calcium channels and NCX are clearly important members of both clocks. In the case of L-type calcium channels, they depolarize the cell following I_f_ activation (membrane clock) but are also a major source of calcium influx to the cell, increasing the calcium loading of the SR and promoting calcium release. This holds both for spontaneous release, which belongs to the calcium clock, as well as release triggered by the calcium influx driven by the L-type current itself. NCX depolarizes the cell following a calcium release, irrespectively of whether the release was spontaneous or triggered. However, the interactions between membrane and calcium clocks are not limited to the L-type calcium channels and NCX. For example, a change in potassium currents, a seemingly evident component of the membrane clock, will alter the duration of the action potential, thus the duration of the calcium influx, and thus the loading of the SR, also affecting the calcium clock. In turn, changes to supposedly pure calcium-related proteins, such as ryanodine receptors or SERCA pumps, will change the cellular calcium transient. This will, in turn, affect the calcium-dependent inactivation of L-type channels [113], and allosterically regulate NCX [114] and influence delayed potassium rectifier current I_Ks_ [115], therefore also affecting the membrane clock. The membrane and calcium clock are further coupled via cAMP and CaMKII signalling, as discussed below.

The existence of two clocks poses the question of whether one is dominant over the other, and this is something that has been debated in the literature [116]. Available evidence suggests that in fact both clocks are equally important, which is perhaps not surprising, given their tight coupling. For the membrane clock, e.g. the experiments by Fenske *et al*. [117] demonstrate how making I_f_ insensitive to cAMP stimulation leads to severe bradycardia and irregular heart rate in mice. With regards to the importance of the calcium clock, experiments using the fast calcium buffer BAPTA have shown that reducing calcium concentration may reduce [118] or even abolish [119] heart rhythm, depending on the BAPTA concentration used.

Below we discuss how proteins involved in membrane and calcium clocks are modulated by cAMP signalling, as well as what are less direct cAMP-related interactions.

## 10. cAMP and membrane clock

The funny current is an integral part of the membrane clock, and is mediated mainly by the HCN4 channels, with minor involvement of HCN1 and HCN2 [120]. The HCN4 function is clearly increased under adrenergic stimulation, but the exact nature of the regulation is not straightforward. Early, as well as more recent studies indicate that cAMP directly binds to HCN channels to stimulate the funny current [121,122]. However, PKA phosphorylation (downstream of cAMP increase) also appears to lead to enhancement of the channel function [123,124]. These two forms of adrenergic enhancement of the funny current are not mutually exclusive, and it is likely that they both take place and possibly involve distinct plasmalemma domains. Consistent with this notion, a study by St Clair *et al*. [125] indicates that PDE3 and PDE4 differentially regulate HCN funny current by controlling separate cAMP domains and by differentially constraining access of the second messenger to HCN channels. Two separate mechanisms seem to be at play: while PDE4 restricts direct access of cAMP to HCN in a domain that does not involve PKA activity, at least at rest, PDE3 appears to control a separate cAMP pool that requires PKA activation to potentiate the channel voltage-dependent gating, both at rest and in response to β-adrenergic stimulation. Intriguingly, a study found that physical association of the HCN4 channels with *β*2-AR is necessary for adrenergic regulation of the funny current [19]. Although the mechanism remains unclear, one could speculate that the association between the channel and the receptor may serve to expose cAMP-binding and PKA phosphorylation sites, which are otherwise inaccessible. This would provide a way of assuring that the bulk of the funny current is under highly local and tight adrenergic regulation, as HCN channels not adjacent to a *β*2-AR would presumably be in the cAMP-insensitive, and thus relatively quiescent, mode.

The L-type calcium current contributes to both the membrane clock (by depolarizing the cell) and the calcium clock (being the main source of calcium influx into the cells). Its pharmacological blockade was observed to reduce the rate of activation of sinoatrial cells [126–128]. The channels carrying the current were long thought to be phosphorylated directly by PKA, but recent studies demonstrated that the cAMP-dependent activation of the current is in fact mediated by Rad, a channel inhibitor associated with the local signalosome [129–131]. The regulatory sequence therefore involves cAMP activation of PKA, phosphorylation of Rad molecules in the vicinity and consequent release of its inhibitory action on L-type calcium current, which is enhanced. While first observed in ventricles, the role of Rad in SAN cells appears to be similar, where an increased resting heart rate following ablation of Rad was observed [132], which is consistent with the L-type calcium current disinhibition and the role of the current in heart rhythm generation.

Another prominent member of the membrane clock which also belongs within the calcium clock, is the NCX. Although PKA does not appear to directly regulate NCX [133], this possibility has not been ruled out entirely [134]. However, the activity of NCX is regulated indirectly during sympathetic activity, via the sodium-potassium pump. The latter is activated by the cAMP pathway [135], via PKA-dependent phosphorylation of the pump-associated inhibitory protein phospholemman, resulting in increased pump activity and reduced intracellular sodium concentration [136]. This consequently biases the NCX to reduce the intracellular calcium concentration, partly compensating for the increased calcium influx via L-type calcium current. This phenomenon was observed in stimulated canine cells [137] and mouse ventricular myocytes where genetic deletion of phospholemman resulted in a greater increase in calcium transient amplitude and in SR calcium loading following sympathetic activation compared to controls [138]. In the context of SAN cells and pacemaking, it would be expected that the lower SR loading following sympathetic activation of the sodium-potassium pump would lead to less spontaneous calcium release and thus would attenuate the heart rate increase following from activation of other members of the calcium and membrane clocks.

## 11. cAMP and calcium clock

SERCA pumps are a crucial contributor to the operation of the calcium clock, refilling the SR with the calcium released. They are under the inhibitory influence of PLB, which is relieved upon PKA phosphorylation of PLB [85,139]. Interestingly, the baseline phosphorylation of PLB (resulting in elevated SERCA function) is much higher in sinoatrial cells than in ventricle [140], probably as a consequence of the overall higher constitutive AC activity of these cells. This has interesting functional implications. The low baseline phosphorylation in the ventricle means that the SERCA function can be substantially increased via PLB phosphorylation, but not reduced. Conversely, the higher baseline PLB phosphorylation in the SAN means that by increasing or reducing the cAMP levels, the sympathetic and parasympathetic nervous system may enhance, as well as reduce SERCA function, correspondingly turning the dial of the calcium clock in either direction. Another source of sinoatrial-specific bidirectional control of the SERCA function is the calcium cycling, which controls the cAMP levels, and thus PLB phosphorylation, via the action of calcium-sensitive ACs [141].

PLB phosphorylation is also tightly controlled by PDEs. Vinogradova *et al*. demonstrated that PDE3 and PDE4 control PLB phosphorylation levels in sinoatrial cells in a synergistic way [84]. Whereas exclusive inhibition of PDE3 or PDE4 lead to about 20% increase in PLB phosphorylation, dual inhibition increased the phosphorylation by about 110%. Similar synergy between PDE3 and PDE4 was observed also for the L-type calcium current [84]. Synergy between two PDEs was noted in multiple cell types and contexts, and its causes are incompletely understood. One possible explanation may be that the higher-cAMP-affinity PDE3 hydrolyses most of cAMP at baseline, but when blocked, the increased cAMP levels may approach the levels that are hydrolysed by the lower-affinity PDE4, which may be further activated by PKA [85].

Ryanodine receptors are another key component of the calcium clock. As mentioned previously, the functional implications of possible phosphorylation by PKA are unclear and controversial [79]. However, there are several indirect ways cAMP signalling may regulate the function of ryanodine receptors: (i) enhanced calcium loading of the SR following SERCA activation promotes their opening, (ii) enhanced calcium influx following L-type channel activation promotes their opening, and (iii) their opening and leakiness is promoted by CaMKII phosphorylation, which itself is stimulated by cAMP signalling, as discussed in the next section.

Inositol 3 phosphate (IP_3_) has also been implicated in the regulation of the calcium clock. IP_3_ is a calcium-mobilizing second messenger generated on activation of G*α*_q_-coupled receptors at the plasmalemma. IP_3_ acts to open IP_3_ receptors (IP_3_R) on the membrane of the SR, releasing calcium in the cytosol. SAN and atrial cells express relatively high levels of IP_3_R compared to the ventricles and IP_3_ is positively chronotropic [142]. The mechanism involved is believed to rely on sensitization of nearby ryanodine receptors by the calcium that is released on opening of IP_3_R, resulting in a larger calcium release through ryanodine receptors in response to opening of L-type calcium channels [143]. However, evidence is emerging that indicates a coupling of IP_3_-dependent calcium release with cAMP signalling, which involves activation of the calcium sensitive AC1 and AC8, a mechanism that is supported by evidence of physical proximity of these cyclases with the IP_3_R [144].

## 12. Other relevant cAMP interactions

Another important signalling pathway in the heart involves CaMKII which serves to integrate information on calcium over time, increasing in activity when calcium transients are frequent. CaMKII is particularly closely connected to the function of cAMP signalling in SAN cells, given the prominence of AC1 in this cell type, which is responsible for the calcium-sensitivity of cAMP production. Pharmacological blockade of CaMKII was observed to lead to a dose-dependent reduction in firing rate in rabbit sinoatrial cells, supporting its importance in the generation of cardiac rhythm [118,145].

Following its activation, CaMKII phosphorylates proteins involved in calcium handling, such as L-type calcium channels and ryanodine receptors, and it activates SERCA pumps, which all contribute to the generation of cardiac rhythm via the calcium clock. CaMKII appears to be tethered to L-type channels directly [146,147], and its activation was reported to lead to a larger peak current and slower current inactivation [148], both increasing the calcium influx to the cell. Phosphorylation of ryanodine receptors by CaMKII facilitates their opening by increasing their calcium-sensitivity and overall opening probability [149,150]. This may be particularly important in the setting of SAN cells, where spontaneous calcium releases drive the calcium clock. SERCA pumps are also suggested to be activated by CaMKII according to a number of studies using pharmacological blockers [151,152], although this is not a universal finding [153]. The exact nature of this regulation is still not fully understood [154] and, unlike PKA phosphorylation, the effects of CaMKII do not seem to involve PLB phosphorylation [155]. Further research into the mechanistic nature of how CaMKII regulates SERCA function is required.

An intriguing recent study in rat ventricular myocytes found that AKAP18*δ*, which assembles the cAMP signalosome around SERCA pumps and ryanodine receptors, also binds and regulates CaMKII, yielding a combined cAMP-CaMKII nanodomain [156]. Interestingly, this study found that the binding of CaMKII to the AKAP at different sites led to either activation or inhibition of CaMKII. The authors propose a working model where CaMKII inhibition prevails at slow heart rates, but as calcium transients become frequent, the CaMKII activity enhancement prevails, providing another layer of complexity to how CaMKII may be activated by calcium signals. Whether this signalosome also operates in SAN cells remains however to be established.

CaMKII activation was suggested to take part in a negative feedback loop of adrenergic activation in the heart, where cAMP-dependent activation of EPAC leads to activation of CaMKII, which in turn activates PDE4D, limiting the cAMP levels that the cell may reach [157]. The importance of this feedback mechanism in sinoatrial cells is yet to be demonstrated, but it is not unlikely, given the prominence of PDE4D observed in rabbit SAN [84].

On activation by EPAC2, CaMKII was also shown to increase the phosphorylation of ryanodine receptors, making them more leaky [158], and to some extent also of phospholamban, potentiating SERCA pumps [159]. To our knowledge, the role of EPAC2 in pacemaker cells has not been studied extensively, but assuming similar function to ventricular cells, by these mechanisms EPAC2 signalling would be expected to enhance the operation of the calcium clock.

Another class of cAMP effectors is the POPDC proteins [32,160]. A study in *Popdc1 and Popdc2* knockout mice reported stress-induced sinus node bradycardia and pauses in heart rhythm [161], a phenotype resembling sick sinus syndrome in patients. POPDC proteins include a cAMP binding domain at their C-terminus and can be phosphorylated on activation of β-ARs [162], but current understanding of the role played by these proteins in pacemaker activity and, in general, in cell physiology, remains limited. One hypothesis is that binding of cAMP or phosphorylation of POPDC proteins may affect their interaction with sarcolemmal components involved in the control of heart rhythm. In support of this idea, NCX1, one of the membrane clock components that functionally couples the membrane clock and the calcium clock, was recently identified as an interactor of POPDC2 [163] and loss of NCX1 causes a SAN disfunction similar to that observed in POPDC mutants [164].

## 13. Conclusion and perspective

cAMP signalling is a critical component of cardiac rhythm regulation as it enhances the activity and helps synchronize the membrane and calcium clocks in the sinoatrial pacemaker cells. Correct function of SAN cells requires coordinated regulation of multiple cAMP subcellular domains, each assembled via protein–protein interactions involving a unique combination of signalling components and each residing at a specific subcellular location.

While a significant amount of information is now available on the role of localized cAMP signalling in healthy pacemaker cells, much remains to be discovered. Key questions that will undoubtedly receive increasing attention and drive future research include the following: what are the details of the molecular machinery that defines compartmentalized cAMP signalling in SAN cells? How does the machinery in SAN cells differ from that in atrial and ventricular myocytes? Does disruption of compartmentalized cAMP signalling contribute to the disturbance of rhythm generation? Also, can defects in cAMP compartmentalization in SAN cells be targeted to treat cardiac disease? The bulk of our knowledge on nanodomain remodelling in disease remains limited to studies in ventricles [13,53,165,166]. Answering these questions will be of critical importance to better understand the pathophysiology of heart disease more widely and for the development of more effective treatments. A recent study investigating the mechanisms of SAN dysfunction in heart failure with preserved ejection fraction uncovered functional, structural and molecular disarray in SAN cells, impacting membrane and calcium clocks and responsible for the reduced chronotropic reserve associated with this condition [167]. Chronotropic incompetence is a frequent cause of exercise intolerance and is a predictor of adverse cardiovascular events and reduced quality of life [168]. Given the vital importance of β-AR signalling in the response to stress and the involvement of compartmentalized signalling in the regulation of membrane and calcium clocks, a better understanding of the role of local cAMP signal remodelling in SAN cells is warranted and may help design tailored therapies in the future.

## Data Availability

This article has no additional data.

## Endnotes

1 Named after the Czech anatomist Jan Evangelista Purkyně. Interestingly, the name is often mispronounced compared to the original pronunciation. Rather than the common ‘purkinzhee’, the correct pronunciation is close to ‘purkinye’. We speculate that the shift in pronunciation arose in the first half of the twentieth century, possibly owing to the article by Hykeš [110] or a similar one, where the Czech writers tried to avoid the letter ‘ě’ not present in most alphabets. ‘Purkyně’ and ‘Purkinje’ are pronounced almost identically in Czech, which is why the authors may have thought the latter to be a good substitute. In addition, it is pronounced similarly to the Czech pronunciation in German, which was the other major academic language in the region in late nineteenth/early twentieth century. However, the pronunciation in English is very different, probably underlying the divergence of pronunciation from the original, despite the fact that Hykeš does suggest how to pronounce the name in his article (‘poorkeynie’).
